# Peer Review of “Why We Are Losing the War Against COVID-19 on the Data Front and How to Reverse the Situation”

**DOI:** 10.2196/29100

**Published:** 2021-05-05

**Keywords:** COVID-19, learning health systems

*This is a peer-review report submitted for the paper “Why We Are Losing the War Against COVID-19 on the Data Front and How to Reverse the Situation”.*

## Round 1 Review

Dear authors and editor, I thank you for the opportunity to review this manuscript [1]. The paper addresses a topic of extreme relevance to public health and economy. This paper is well established, and the subject is interesting, but some major revisions should be considered.

1. It is hard to follow how future patterns can be predicted as enterprises navigate new corporate social responsibility strategies through the epochal challenges presented by COVID-19.

2. The abstract should briefly state the purpose of the research, the principal results, and the major conclusions. An abstract is often presented separately from the article, so it must be able to stand alone.

3. The necessity and innovation of the paper should be presented in the *Introduction* section.

4. The structure of the paper should be presented at the end of the *Introduction* section.

5. Here are some new, related references, which should be added to the reference list: Khorram-Manesh et al [2] and Purngagen et al [3].

6. Please ensure your conclusions underscore the scientific value added by your paper, and/or the applicability of your findings/results, as indicated previously. Please revise the conclusion section to include more details. Basically, you should enhance your contributions and limitations, underscore the scientific value of your paper, and/or discuss the applicability of your findings/results and future study in this section.
